# Layered Double Hydroxide Fluoride Release in Dental Applications: A Systematic Review

**DOI:** 10.3390/dj7030087

**Published:** 2019-09-02

**Authors:** Agron Hoxha, David G. Gillam, Andy J. Bushby, Amani Agha, Mangala P. Patel

**Affiliations:** 1 Oral Bioengineering, Barts and the London School of Medicine and Dentistry, Institute of Dentistry, Queen Mary University of London, Mile End Road, London E1 4NS, UK; 2 Centre for Adult Oral Health, Barts and the London School of Medicine and Dentistry, Institute of Dentistry, Queen Mary University, New Road, London E1 2AD, UK; 3 School of Engineering and Materials Science, Queen Mary University of London, London E1 4NS, UK

**Keywords:** layered double hydroxide, fluoride, dentistry, systematic review

## Abstract

This systematic review appraises studies conducted with layered double hydroxides (LDHs) for fluoride release in dentistry. LDH has been used as antacids, water purification in removing excess fluoride in drinking water and drug delivery. It has great potential for controlled fluoride release in dentistry, e.g., varnishes, fissure sealants and muco-adhesive strips, etc. The Preferred Reporting Items for Systematic Reviews and Meta-Analyses (PRISMA) Statement was followed with two reviewers performing a literature search using four databases: PubMed, Web of Science, Science Direct and Ovid Medline with no date restrictions. Studies including any LDH for ion/drug release in dentistry were included, while assessing the application of LDH and the value of the methodology, e.g., ion release protocol and the LDH production process. Results: A total of 258 articles were identified and four met the inclusion criteria. Based on two in vitro studies and one clinical study, LDH was previously studied in dental materials, such as dental composites and buccal muco-adhesive strips for fluoride release, with the latter studied in a clinical environment. The fourth study analysed LDH powder alone (without being incorporated into dental materials). It demonstrated fluoride release and the uptake of volatile sulphur compounds (VSC), which may reduce halitosis (malodour). Conclusion: LDHs incorporated in dental materials have been previously evaluated for fluoride release and proven to be clinically safe. LDHs have the potential to sustain a controlled release of fluoride (or other cariostatic ions) in the oral environment to prevent caries. However, further analyses of LDH compositions, and clinical research investigating any other cariostatic effects, are required.

## 1. Introduction

A wide range of materials have been extensively investigated to obtain a slow and controlled drug/ion release, especially for applications within medicine and dentistry. Within dentistry, it is acknowledged that obtaining a low-level release of fluoride in the oral environment is essential, as it is the most effective method for preventing post-eruptive dental caries. The physiological fluoride concentration in saliva varies between individuals and ranges from 0.02 to 0.05 ppm; however, numerous studies have demonstrated a decrease in demineralisation, with an increase in fluoride concentration (0.025–2 ppm) [1,2,3]. Fluoride inhibits mineral loss (via forming a less soluble fluorapatite) and enhances remineralisation of enamel or decalcified dentine. It also has the ability to inhibit the metabolism and growth of bacteria, including *Streptococcus (S.) mutans* and *S. sobrinus* that initiate dental caries [4,5,6].

Healthcare companies provide dentists with fluoride-releasing dental materials, such as glass ionomer cements (GIC), but the release is not controlled and diminishes over time [7,8]. Also, topical fluoride applications via toothpastes, mouthwashes and fluoridated water, used globally to prevent caries, do not maintain a low-level delivery of fluoride. High levels (e.g., above 11 ppm) are toxic, cause fluorosis, and do not reduce enamel demineralisation [9,10,11,12]. Therefore, a material with a controlled and prolonged delivery of fluoride is required in dentistry to help inhibit/prevent caries.

Layered double hydroxides (LDHs) are capable of anion exchange and have thus attracted attention as promising functional materials for a number of applications, including water purification. LDHs, also known as hydrotalcites, consist of positively charged metal sheets, thus producing a layered structure, which provides a large surface area, as well as a high anion exchange capacity [13]. LDHs have been successfully proven to remove excess fluoride from drinking water [14] and are biocompatible, having been studied in biological applications for controlled drug release systems [13,15,16]. For example, a non-ionic and poorly water-soluble anti-cancer drug, 10-hydroxycamptothecin, was successfully encapsulated using LDH and liposomes for a controlled drug delivery with a good water dispersity (not allowing aggregation) [17]. Furthermore, LDH is commercially available in the form of antacids and antipeptics, such as Talcid™ and Altacite™, respectively [13].

**Figure 1:**
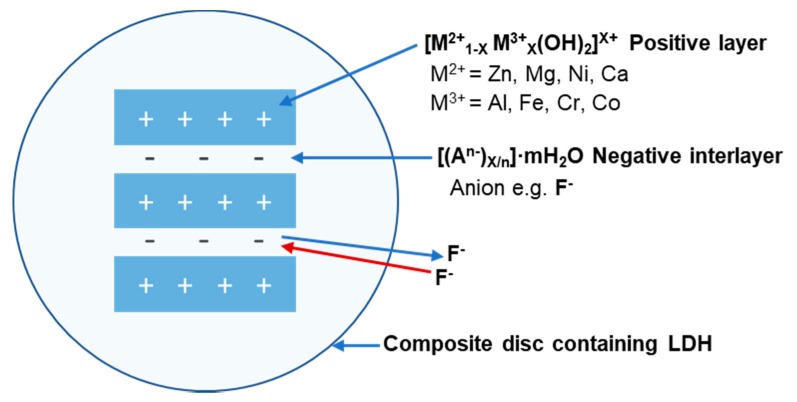
Schematic illustration of an enlarged LDH structure incorporated into, e.g., a composite material. The structure of the positive layer and negative interlayer displays fluoride absorption and release.

The general formula for LDH is [M^2+^_1−X_ M^3+^_X_(OH)_2_][A^n−^_X/n_· mH_2_O] consisting of divalent M^2+^ (Mg, Zn, Ca, Ni, Mn, etc.) and trivalent M^3+^ (Al, Cr, Fe, V, Co, etc.) cations. A range of anions (A^n−^), such as F^−^, Cl^−^, NO_3_^−^, CO_3_^2^^−^, SO_4_^2^ and organic anions, can be used as intercalating anions between the positive sheets (Figure 1) [18].

The additional benefit of LDH is that the divalent or trivalent ion may be altered during synthesis. Therefore, further benefits from specific cations can also be achieved in dentistry, e.g., release of calcium ions from the LDH structure itself could produce hydroxyapatite, or zinc ions may further reduce demineralisation [19,20]. Also, the formation of hydroxyapatite or fluorapatite may contribute to the reduction in dentine hypersensitivity (DH), as demonstrated with ion-releasing bioactive glasses [21,22]. This is a novel area for LDH, and the smaller sized crystals of LDH may be used to occlude dentine tubules to treat DH more effectively, in contrast to larger bioactive glass particles currently used via toothpastes/mouthwashes [21,22]. Bioactive glasses comprise a network of, for example, silicon dioxide, phosphorus pentoxide, calcium oxide, sodium oxide and fluorite (SiO_2_-P_2_O_5_-CaO-Na_2_O-CaF_2_). Those containing fluoride release it upon dissolution of the glass [23]. In contrast, LDH consists of a non-soluble layered structure, which is able to absorb and release fluoride from the interlayer spaces within the matrix [24]. Therefore, the LDH structure will remain unchanged in the dental material, e.g., dental composite and/or toothpaste/mouthwash. Another advantage of using LDH is that it is inexpensive and easy to synthesize through a co-precipitation method, even though some impure forms exist naturally.

In relation to dentistry, there is limited research on the use of LDH. Therefore, the current systematic review aims to identify and understand any previous research conducted with the use of LDH in dentistry.

## 2. Methods

### 2.1. Research Question

A research question was formulated prior to conducting a comprehensive systematic review: “Have layered double hydroxide(s) previously been studied for application in dentistry/clinical studies, as an active ingredient to prevent caries?”

### 2.2. Search Methodology

Four individual electronic databases were analysed to identify the relevant articles published in accordance with the search strategy, Preferred Reporting Items for Systematic Reviews and Meta-Analyses (PRISMA) statements for assessing the methodological quality of systematic reviews [25]. The databases searched (January 2018) included PubMed, Web of Science, Science Direct, and Ovid Medline for articles with no date restrictions.

The keyword combination used in searching the database were: (layered double hydroxide OR hydrotalcite) AND (release) AND (fluoride)).

The search strategy for MEDLINE via OVID:

1. Layered double hydroxide
2. Hydrotalcite
3. Release
4. Discharge
5. Fluoride
6. Fluoride ion
7. Fluoridation
8. 1 OR 2
9. 3 OR 4
10. 5 OR 6 OR 7
11. 8 AND 9 AND 10.

### 2.3. Study Selection

The titles and abstracts of all articles were searched and read using the selected keyword combination presented above, with any duplicates being identified and removed from the total list. This was conducted by three authors: Agron Hoxha (AH) and Amani Agha (AA), with Mangala Patel (MP) as the final arbitrator. If the information from the titles and abstracts obtained from the initial search were unclear regarding whether the inclusion criteria were met, then a full text of the article was obtained to identify its suitability based on the inclusion and exclusion criteria for the review (Table 1). The requirements for inclusion were discussed by two investigators (AH and AA) and discussed with MP prior to final acceptance or rejection.

### 2.4. Study Quality Assessment

The quality of each selected paper was also evaluated by two investigators (AH and AA), according to: LDH preparation, LDH characterisation, fluoride release, cytotoxicity, statistical analysis, incorporated into a dental material and whether a control group was used. The risk of bias was assessed using the Cochrane risk of bias tool with ROBINS-I [26]. The quality of each paper was “graded” in terms of high, medium, or low quality, depending on the number of parameters met by ‘’Yes’’ on each paper or ‘’No’’ if the parameter was not met (Table 2). The scoring was as follows: low quality if 1–3 scored ‘’Yes’’; medium quality if 4 or 5 scored ‘’Yes’’; high quality if 6 or 7 scored ‘’Yes’’. If a lack of homogeneity was present with the identified studies, then a meta-analysis will not be considered.

## 3. Results

### 3.1. Study Selection

**Figure 2:**
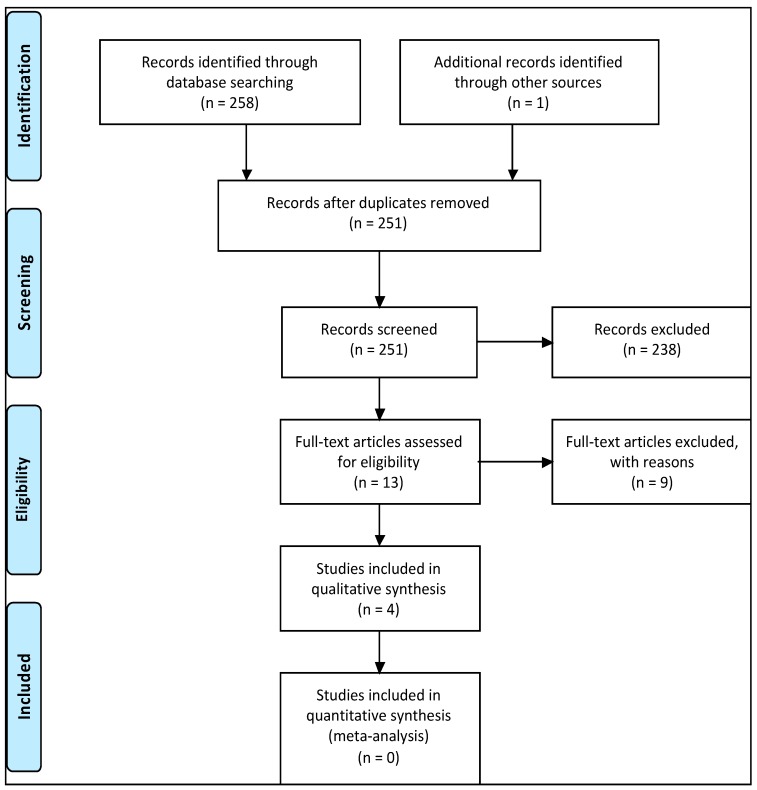
A flowchart of the search strategy in accordance with the PRISMA strategy.

The initial electronic search using the keywords used above identified 258 articles. One article was found via a symposium program, which was also published in a peer-reviewed article (Key Engineering Materials). This article was not identified via the online database search. After excluding duplicates, 251 articles remained (Figure 2). After screening the title and abstracts of the remaining articles, 238 articles were removed for not meeting the set inclusion criteria. A detailed full-text review was performed on the 13 remaining articles, where a further nine were removed (Table 3) due to failure to meet the inclusion criteria. One clinical study and three non-clinical articles were identified during the search and included in this review.

### 3.2. Study Quality Assessment

Of the four studies included, three were graded as high quality and one as low quality depending on the parameters met, as demonstrated in Table 2. However, all studies demonstrated that fluoride release was investigated, which was of importance for this review.

### 3.3. Study Characteristics

#### 3.3.1. LDH—Polymer Composition and Study Characteristics

Two of the articles analysed the properties of the same LDH composition: a nitrate version of MgAl LDH, [Mg_0.65_Al_0.35_(OH)_2_](NO_3_)_0.35_·0.68H_2_O [27,28]. The nitrate-containing powder was charged with fluoride via stirring for 48 h in 0.25 M of sodium fluoride (NaF) solution to potentially form [Mg_0.65_Al_0.35_(OH)_2_](F)_0.35_·0.8H_2_O, via anion exchange. Both of these studies incorporated the nitrate version MgAl LDH powders into commercial light-activated restorative material (Kerr s.r.l., Salerno, Italy) containing bisphenol-A glycidyl dimethacrylate (Bis-GMA), tri-ethylene glycol dimethacyrlate (TEGDMA), camphorquinone (CQ), ethoxylated bisphenol A dimethacrylate (EBPADMA) and glass filler [27,28]. Perioli et al. [30] also used a similar LDH composition containing MgAl as a nitrate version; however, the powder was charged with a 0.2 M NaF solution for 48 h rather than 0.25 M NaF. This LDH powder was, however, incorporated into a muco-adhesive polymer (Table 4) rather than a light-curable composite material. The final LDH powder formula was proposed as being [Mg_0.63_Al_0.37_(OH)_2_](F)_0.36_(NO_3_)·0.8H_2_O. Yokogawa et al. [29] used a different LDH composition, using iron (Fe) as the trivalent metal ion rather than Al. The LDH composition (MgFe) contained a higher ratio of divalent to trivalent ions (2.7:1), with the final formula proposed as being [Mg_0.73_Fe_0.27_(OH)_2_](F)_0.35_·0.2H_2_O. However, the fluoride absorption concentration and time of absorption were not mentioned in this study. Prior to charging with fluoride, the LDH was calcined to 500 °C. This procedure was not performed in the other studies.

#### 3.3.2. Characterisation Techniques

The included studies used a wide range of characterisation techniques (Table 4) and investigated various properties for different applications. Two of the studies investigated the effect of a fluoride-releasing material, with a focus on cell behaviour [27,28]. Perioli et al. [30] investigated fluoride release (in vitro), from a buccal muco-adhesive strip containing LDH, and also assessed its biocompatibility (in vivo) on five healthy volunteers’ gums. Another study focused on the release of fluoride and the adsorption of volatile sulphur compounds (VSC) from the LDH powder alone, with no matrix, as in the other studies [29].

X-ray powder diffraction (XRPD) was used to examine the structure of LDH and to confirm that LDH was formed [28,29,30]. Scanning electron microscopy (SEM) was used to examine the morphology of the LDH powder and to view any fractures on the buccal adhesive film [29,30]. The adhesive strength of the strips was assessed with a dynamometer using porcine mucosa [30]. Energy dispersive X-ray spectroscopy (EDX) and inductively coupled plasma-optical emission spectroscopy (ICP-OES) were used to analyse the elemental composition and ratio/content of divalent and trivalent ions within the LDH powder [29,30]. Mechanical properties of the composite materials were analysed using dynamic mechanical analysis (DMA) by applying a variable flexural deformation in a dual cantilever [27,28]. The influence of fluoride release on cell behaviour (human dental pulp stem cells) were analysed using a range of techniques, as shown in Table 4. However, the methodological procedure of such investigations was not the focus of this systematic review. A summary of the results from each identified study are given in Table 4.

#### 3.3.3. Fluoride Release Protocol and Data

The LDH powders used in the four studies were all charged with different concentrations of fluoride, i.e., 0.25 M of NaF solution for 48 h and 0.2 M for 48 h [27,28,30]. Also, a range of release media was used for each study, e.g., artificial saliva, physiological saline solution (NaCl 0.9% *w*/*v*), hydrogen sulphide (H_2_S), water and de-ionised water containing 1.2 mM NaHCO_3_. All studies were conducted at 37 °C; however, Yokogawa et al. [29] did not specify at which temperature the study was conducted.

**Figure 4:**
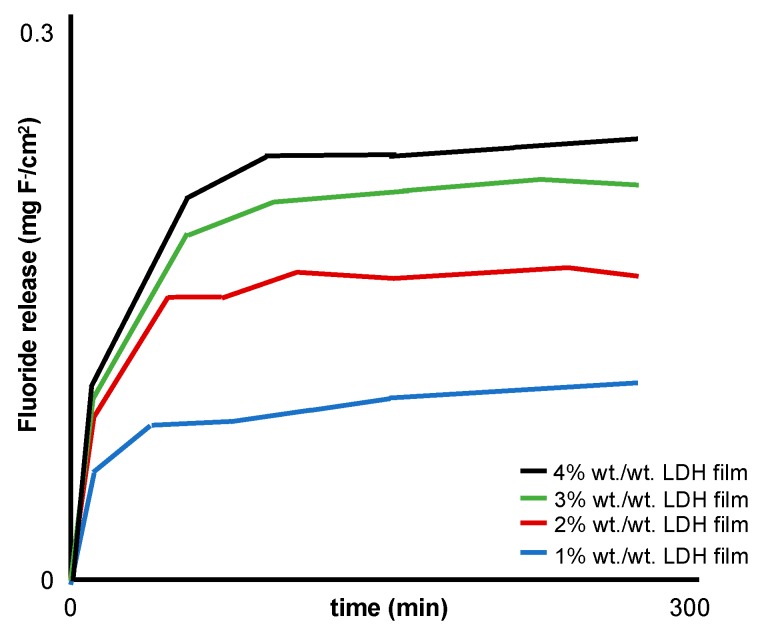
Modified schematic from Perioli et al. [30] representing the in vitro fluoride release profiles from a buccal muco-adhesive strip containing 1–4% *w*/*w* MgAl LDH.

Various techniques were used to measure the concentration of fluoride in the release media: ion chromatography, ion-selective electrodes (ISEs) and ultraviolet–visible spectroscopy [27,28,29,30]. All the studies demonstrated the ability of LDH to release fluoride, e.g., Perioli et al. [30] reported an increase in fluoride release with an increase in LDH loading (1–4% *w*/*w*; Figure 4).

## 4. Discussion

In relation to dentistry, there is limited research on the use of LDH. Therefore, this systematic review was conducted to identify and examine previous research focusing on ion release from LDH in the field of dentistry and to evaluate possible future research opportunities for the use of LDH in this area. LDH appears to be relatively new to research in the dental field. Only four studies were identified based on the set inclusion criteria, which included LDH in dental applications. Thus, at this early stage of LDH research in dentistry and with a wide range of LDH compositions available (as mentioned in the introduction), investigating the composition of LDH is vital in order to understand its fluoride-release properties. However, from the four studies included, three used a magnesium-containing LDH (MgAl-LDH) at a 2:1 ratio [27,28,30], and one study used MgFe-LDH (trivalent ion = Fe and not Al) at a 2.7:1 ratio [29]. Other compositions of LDH can be utilised with varying divalent (e.g., calcium and zinc etc.) to trivalent cation ratios (1:1, 2:1 and 3:1), which could alter the fluoride release rate in order to obtain the optimum therapeutic level for the desired dental application [40].

Fluoride release was demonstrated in all four studies, but in different media, such as artificial saliva, physiological saline solution (NaCl 0.9% *w*/*v*), H_2_S water and 1.2 mM NaHCO_3_. Lv et al. [41] reported that fluoride absorption can be reduced by ≈50% in the presence of other ions, e.g., phosphate, sulphate, nitrate and chloride ions, which in turn will affect the amount of fluoride released by the material. This could also be true for the release of fluoride from LDH, although this has not been described in the literature to date. However, el Mallakh and Sarkar [42] reported that three types of dental glass ionomer cements released either more or less fluoride ions in de-ionised water compared to artificial saliva. Therefore, this suggests that the amount of fluoride released varies in different media, and so fluoride ion release from LDH in different media needs to be calibrated with a range of LDH ratios in order to find the optimum formulation for use in various dental materials.

In addition, investigations on ion-exchange mechanisms correlated to LDH structure also indicate that different media would affect the release of fluoride. Research conducted by Miyata [43] demonstrated that LDHs have different ion-exchange equilibrium constants for the different anions in solution. For example, the ion-exchange equilibrium constants for MgAl-LDH are in the order of OH^−^ > F^−^ > Cl^−^ > Br^−^ > I^−^, and these increase with decreasing basal spacing within the LDH structure. Order of anion-LDH selectivity: NO_3_^−^ < Br^−^ < Cl^−^ < F^−^ < OH^−^ < MoO_4_^2−^ < SO_4_^2−^ < CrO_4_^2−^ < HAsO_3_^2−^ < HPO_3_^2−^ < CO_3_^2−^.

Therefore, a media containing NaHCO_3_ would contain CO_3_^2−^ ions, as reported in the included study by Perioli et al. [30]. One could predict that fluoride release would increase due to CO_3_^2−^ having a higher affinity and therefore displacing the fluoride ions more readily. Hence, the four included studies cannot be directly compared for the rate of fluoride release for each material, e.g., dental composite and buccal muco-adhesive. Since it is also difficult to mimic the dynamics of saliva in the oral environment in vitro, in order to investigate the cariostatic effect of LDH, most studies appear to use a Tris buffer or artificial saliva. These media mimic the oral environment closer than deionised water, and therefore should be recommended for future studies.

Another factor that can affect the release of fluoride is the solution it is charged with. The affinity of the anions within the LDH and the type of fluoride charging solution, for example, sodium fluoride, sodium monofluorophosphate, stannous fluoride or acidulated phosphate fluoride, may affect the amount of fluoride absorbed by the LDH. This will subsequently affect the amount of fluoride released from the LDH. Therefore, further studies on fluoride absorption and release in differing fluoride charging solutions are essential for use of LDH within the dental field.

The preparation technique used for LDH is another factor that may affect fluoride release; for example, only one study (by Yokogawa et al. [29]) from the four included calcined the LDH powder at 500 °C prior to fluoride absorption. It has previously been demonstrated that a thermal treatment of LDH at 450–500 °C increased fluoride absorption by ≈21%. This was partially due to an increase in the specific surface area of the LDH as a result of CO_3_^2−^ being removed, creating vacancies within the positive metal LDH sheets [44]. Future studies investigating the effect of calcination are required as this could possibly prolong the release of fluoride ions to greater than 160 days [28].

Fluoride ion release reported from all the included studies was conducted in a static solution, which results in a buildup of ions in solution until saturation is reached, after which, no further ions leach. The disadvantage of this methodology is that it does not consider the dynamic salivary flow in the oral cavity, which ranges between 0.3 mL/min (unstimulated) and 0.52–4.55 mL/min (stimulated) [44,45,46,47,48]. A study compared the release of fluoride from GICs in continuous flowing (0.5 mL/min rate and F^−^ measurement taken at 1 day and 7 days) and static solutions (solution replaced at 1 day and 7 days). Significantly, low levels of fluoride were reported in the continuous versus static systems (F^−^ release, day 1: 0.05 vs. 0.56 ppm, and day 7: 0.02 vs. 0.16 ppm, respectively) [49]. Therefore, release of fluoride ions should be conducted by both methods in future studies in order to analyse whether a desired therapeutic level of fluoride release for dental applications has been achieved.

The benefits of a slow release of fluoride were reported in vitro by two of the studies in terms of: (1) inducing cell migration, and (2) cell differentiation into the dental hard tissue-forming cells [27,28]. These observations would therefore indicate that future in vivo studies are safe to conduct as the release of fluoride from the LDH was not at a toxic level. In vivo fluoride release studies from LDH have not been reported to demonstrate its benefits; however, in a short clinical study, Perioli et al. [30] reported the tolerability of an adhesive strip incorporating LDH in vivo with respect to residence time, fragment loss, swelling and saliva variations, irritation and pain. This study did not report, for example, any in vivo benefits of fluoride on the enamel surface or the elevated concentrations of fluoride within the oral cavity. Benefits following the release of divalent cations from LDH, e.g., calcium and zinc, which could induce a further cariostatic effect in the mouth, has not been reported and merits further investigation.

The current array of studies (incorporating LDH) identified for this systematic review did not demonstrate the benefit of a controlled release of fluoride on hard tissue (enamel or dentine), such as a reduction in demineralisation via the formation of fluorapatite, or the enhancement of remineralisation. Nevertheless, the studies included in this systematic review demonstrate that LDH is a promising, versatile material with a great potential to act as a slow-releasing vehicle for the prevention of dental caries and malodour, via fluoride release and absorption of VSC, respectively. However, further well-conducted studies are required to substantiate this statement.

Regarding the mechanical properties of dental resins incorporating LDH as a filler, there was evidence from two studies that there was an increase in the mechanical properties, which were analysed using DMA. However, the test specimens were only investigated prior to fluoride release and not after the study period of 160 days. Measuring this property after the study period may have had an adverse effect on the material’s properties [27,28]. It is acknowledged that LDH absorbs water, as well as other anions within the interlayer space, which may cause the dental material to swell. Several studies with LDH-hydrogel nanocomposites have indicated an effect on physical properties due to water absorption [40,41]. Analysing the mechanical properties of hydrated LDH-dental resin specimens is therefore essential for understanding the overall effect of water uptake and fluoride ion release. The LDH’s particle size and distribution could also drastically influence ion release and possibly have a detrimental effect on the mechanical properties. Since the four studies did not investigate the aforementioned LDH properties, these should be explored further.

A large range of research using LDH in the dental field appears promising for future studies, which could vastly improve fluoride release properties obtained from fluoride-glass-filled resins and GICs [7,8,27]. Clearly, further research investigating the incorporation of LDH in other dental materials, for example, dental varnishes and orthodontic cements, is required to show any potential cariostatic effects in the mouth.

## 5. Conclusions

In conclusion, this review only found four papers that met the criteria, where a range of techniques were used to investigate some properties of LDH for potential use in the dental field. These included LDH’s incorporation into a buccal muco-adhesive strip to release fluoride as a powder alone (without incorporating into a dental material) and two studies where it was incorporated in dental resins as a filler. The release of fluoride from LDH in five healthy volunteers was shown to be safe to use in vivo. Therefore, as a result of this systematic review, it is clear that further research on the use of LDH is necessary within the field of dentistry, which examines the controlled release of fluoride (and other beneficial ions) from LDH powders alone and after incorporating them in dental materials. In particular, the effect of released ions on inducing remineralisation and decreasing demineralisation of dental hard tissue is of significant importance.

## Figures and Tables

**Figure 3:**
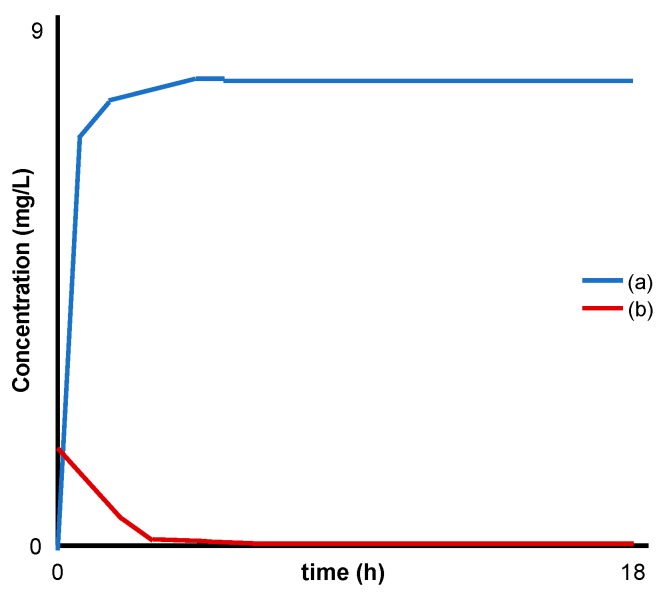
Modified schematic from Yokogawa et al. [29] representing: (**a**) release of fluoride and (**b**) absorption of sulfur compounds (H_2_S water) by MgFe LDH.

**Table 1:** Inclusion and exclusion criteria used for identifying papers in the systematic review.

**Inclusion Criteria for Studies Included**
Investigated any form of layered double hydroxide, for example MgAl, ZnAl, CaAl and MgFe, in dentistry. Reported data on any type of ion or drug release from LDH (e.g., fluoride, calcium, chlorhexidine) in dentistry. Utilised a range of characterisation techniques. Investigated the use of LDH with a potential for dental applications. Any randomised or quasi-randomised clinical studies investigating LDH.
**Exclusion Criteria**
Studies investigating the uptake/removal of ions or drugs by LDH in medicine or non-dental research. Applications of ion release not used in the dental materials market.

**Table 2:** Methodological quality of the selected studies.

Study	LDH Synthesis	LDH Characterization	F^−^ Release	Cyto-Toxicity	Statistical Analysis	Incorporated into Dental Material	Control Group	Grade
Calarco et al. [27]	Yes	No	Yes	Yes	Yes	Yes	Yes	High
Tammaro et al. [28]	Yes	Yes	Yes	Yes	Yes	Yes	Yes	High
Yokogawa et al. [29]	Yes	Yes	Yes	No	No	No	No	Low
Perioli et al. [30]	Yes	Yes	Yes	No	Yes	Yes	Yes	High

**Table 3:** **Nine** studies excluded from the review.

Studies	Reason(s) for Exclusion
Sarijo et al. [31]	Ion release investigated for herbicide release, not release from dental materials.
Saha et al. [24]	Review on drug release; however, not dental related.
Kuthati et al. [32]	Review on drug release reporting only one dental study by Tammaro et al. [28], which is included in our review.
Mandal et al. [33]	Fluoride removal investigated.
Kameda et al. [34]	Fluoride removal investigated.
Delorme et al. [35]	Fluoride uptake and release investigated for water depollution.
Joshi et al. [36]	Layered double hydroxide not used in the study, mesoporous hectorites used.
Louvain et al. [37]	No fluoride release data published; the study was not dentally related.
Ma et al. [38]	Fluoride removal investigated.

**Table 4:** Characteristic details and summary of results from studies identified in the systematic review.

Author(s)	LDH Type and Ratio M^2+^:M^3+^	LDH Incorporated in:	Fluoride Release Protocol	Outcome/Analysis Technique	Summary of Results
Calarco et al. [27]	MgAl 2:1	Composite: UDMA, Bis-GMA, TEGDMA, EBPADMA, glass filler	Discs (14 × 1 mm) in artificial saliva (15 mL at 37 °C). F^−^ release measured per h until 8 h, then daily (10 days), and then weekly (3 weeks).	F^−^ release: ion chromatography Mechanical properties: dynamic-mechanical analysis (DMA) Cytotoxicity assay: MTT Cell migration: modified Boyden chamber method [39]. Odontogenic-related gene expression: polymerase chain reaction.	LDH-fluoride containing dental resins demonstrated: A lower release rate of fluoride compared to fluoride-glass filled dental resins (FGDR). Continuous low release of fluoride increased the migratory response of human dental pulp stem cell subpopulation (STRO-1^+^) and indicated a complete odontoblast-like cell differentiation. Note, this effect was not observed with FGDR.
Tammaro et al. [28]	MgAl 2:1	Composite: UDMA, Bis-GMA, TEGDMA, EBPADMA, glass filler	Discs (20 × 1 mm) in NaCl 0.9% *w*/*v*, 50 mL (37 °C). F^−^ release measured per h (6 h), then 12 h and other intervals (160 days).	F^−^ release: ISE Characterization: XRPD, FTIR, DMA hDPSC proliferation assay: PicoGreen dsDNA and microplate reader Alkaline phosphatase activity Extracellular matrix mineralisation: Alizarin red S staining	LDH-fluoride in dental resins (0.7, 5, 10, 20 wt.%): Improved the mechanical properties with an increase in filler concentration. Released fluoride slowly over 6 months. Increased alkaline phosphatase activity of hDPSCs cells.
Yokogawa el al [29]	MgFe 2.7:1	Analysed LDH powder alone	0.1 g immersed in H_2_S water (300 mL) F^−^ release measured at 1, 2, 3, 4, 5, 6, 12 and 18 h (°C not stated).	F^−^ release: UV-VIS spectroscopy H_2_S uptake: GC/FPD Characterization: XRPD, FTIR, SEM and EDX, particle size analysis	LDH-fluoride was able to uptake volatile sulphur compounds (VSC) and release fluoride (Figure 3). No iron cations were released from the LDH structure.
Perioli et al. [30]	MgAl 2:1	Muco-adhesive patches: sodium carboxy methyl cellulose, polycarbophil propylene glycol, de-ionised water	Circular films (diameter 25 mm) adhered to a Teflon cell with 100 mL of 1.2 mM NaHCO_3_ water (37 ± 0.1 °C) agitated at 60 rpm. F^−^ release in vitro at predetermined times for 4 h.	F^−^ release: ion chromatography Characterization: XRPD, ICP-OES, TGA Film morphology: 8 MP camera and SEM Water holding: weight as produced, after hydration and dehydration Ex-vivo muco-adhesion: dynamometer In-vivo tolerability: five volunteers to evaluate residence time, swelling capacity, salivary modification, fragment loss, acceptability and organoleptic properties.	LDH-fluoride (1–4% *w*/*w*) in a hydrophilic buccal mucoadhesive (2 cm^2^) attached to the gum of five healthy volunteers: Released fluoride at a controlled rate, which increased with an increase in LDH-fluoride. Kinetic studies demonstrated that the concentration gradient of fluoride was the driving force for release. Fluoride release followed Fickian diffusion and a zero-order mechanism

Note: Urethane di-methacrylate (UDMA); bisphenol-A glycidyl dimethacrylate (Bis-GMA); Triethylene glycol dimethacrylate (TEGDMA); ethoxylated bisphenol A dimethacrylate (EBPADMA; Ion selective electrode (ISE); X-ray Powder Diffraction (XRPD); Fourier Transform Infra-Red Spectroscopy (FTIR); Human dental pulp stem cells (hDPSC); Double stranded deoxyribonucleic acid (dsDNA); Gas Chromatography-Flame Photometric Detector (GC/FPD); Scanning Electron Microscopy (SEM); Energy Dispersive X-ray Spectroscopy (EDX); hydrogen sulphide (H_2_S); Inductively Coupled Plasma-Optical Emission Spectroscopy (ICP-OES); Thermal Gravimetric Analysis (TGA); sodium bicarbonate (NaHCO_3_).
